# Xiaoyao-san, a traditional Chinese herbal formula, for the treatment of irritable bowel syndrome: A protocol for a systematic review and meta-analysis

**DOI:** 10.1097/MD.0000000000024019

**Published:** 2021-03-12

**Authors:** JiHo Lee, Won-Suk Sung, Eun-Jung Kim, Young Woo Kim

**Affiliations:** a School of Korean Medicine, Dongguk University, Goyang-si; b Department of Acupuncture & Moxibustion, Dongguk University Bundang Oriental Hospital, Seongnam-si, Gyeonggi-do, Republic of Korea.

**Keywords:** Chinese herbal formula, irritable bowel syndrome, meta-analysis, protocol, systematic review, Xiaoyao-san

## Abstract

**Background:**

Irritable bowel syndrome (IBS) is a disorder which has considerable effect to patient's quality of life and social functioning. Its main symptoms include recurrent abdominal pain and/or bloating associated with abnormal stool form or frequency. The recommendable treatment of IBS is a medication including loperamide, cimetropium, tricyclic antidepressants, and selective serotonin receptor inhibitors, but it has limited effects and several side effects dissatisfy IBS patients. As an alternative therapy, Xiaoyao-san (XYS) is gaining interest for IBS patients. XYS, a traditional Chinese medicine (TCM), has wide scope of indications and it can be prescribed for various gastrointestinal disorders in TCM syndromes but there has been no systematic review on IBS. Therefore, this review aims on systematically validating the curative effect of XYS on IBS.

**Methods:**

Electronic databases, manual search, and contact to author e-mail will be used for searching randomized controlled trials about the use of XYS for IBS. We will select studies by the predefined criteria and collect the data on study participants, interventions, control groups, outcome measurement, adverse events, and risk of bias. Primary outcome will be the efficacy rate, and secondary outcomes will be the IBS-centered indices (abdominal pain score, abdominal distension score, diarrhea or constipation score, bowel symptom severity scale), index about quality of life, and adverse events. Review Manager software and Cochrane Collaboration “risk of bias” tools will be used for meta-analysis and assessment of risk of bias.

**Results:**

This review will identify the clinical evidence of XYS's effectiveness and safety for IBS according to formal evaluation aspects.

**Conclusion:**

This review will further support the evidence-based usage of XYS for IBS treatment.

**Ethics and dissemination:**

No ethical approval is required since there is no personal information collection and patient recruitment.

**Trial registration number:**

Research Registry; reviewregistry986.

## 1 Introduction

Irritable bowel syndrome (IBS) is a functional gastrointestinal disorder which considerably affects patient's quality of life and gives economic burden to society.^[1]^ Main symptoms of IBS include recurrent abdominal pain and/or bloating associated with abnormal stool form or frequency without identifiable biological markers.^[1,2]^ The pathogenesis of IBS is considered to be an alternation in brain-gut interactions which is related with gut motility, visceral hypersensitivity, and abnormal autonomic gastrointestinal responses,^[3]^ and there are also variable risk factors including genetics, diet, gut microbiome, geography, and culture.^[4]^

Regarding the classification, the Rome criteria divided IBS into 3 subtypes (IBS-D, IBS-C, and IBS-M) according to the main-symptoms and each distribution is known to be as follows; 46% of IBS-D, 32% of IBS-C, and 22% of IBS-M.^[5]^ The prevalence of IBS usually varies between 5% and 20% in accordance with the region and the diagnostic criteria.^[6]^ In Korea, 6.0% of population suffered from IBS and national health insurance costs was estimated to be about $155 million.^[7]^ The prevalence of IBS in the United States of America was 4.7% according to Rome IV(8.6% in Rome III) and 10% to 15% in Europe.^[8,9]^ The economic burden of these countries was estimated to be $10 billion in the United States of America and €8 billion in Europe.^[10,11]^

The recommendable treatment for IBS is medication. Loperamide, cimetropium is considered as the first-option and tricyclic antidepressants, selective serotonin receptor inhibitors, lubiprostone, rifaximin, pregabalin is used for secondary options.^[1,12]^ Although these medicines have clear curative effect on IBS symptoms, limited and temporal effects as well as side effects remain to be a problem, resulting in not fully satisfying IBS patients.^[13]^ Psychological therapies are also considered for the management of IBS but its efficacy remains unclear.^[14]^

Due to the limitations of conventional treatments, IBS patients have been tried to seek for complementary and alternative therapies including yoga, biofeedback, osteopathic manipulation, and massage.^[15]^ Traditional Chinese medicine (TCM), one of the complementary therapies, has shown its therapeutic effect using acupuncture,^[16]^ herbal formula,^[17]^ moxibustion,^[18]^ and tuina.^[19]^

Xiaoyao-san (XYS) is a herbal formula which has wide scope of indications related to liver stagnation in TCM syndrome. There are some conducted or ongoing reviews for XYS in treating psychological disease, hypertension and functional dyspepsia.^[20–23]^ According to gastrointestinal disorders, XYS can be applied to abdominal pain, abdominal distention, diarrhea or constipation.^[24]^ Clinical trials indicating the benefits of XYS for IBS has been constantly published, but no reviews covering the effectiveness and safety of XYS for IBS are published in English. Therefore, it is necessary to identify the possibility of XYS in the management of IBS.

This systematic review aims on systematically validating whether XYS has curative effect on IBS comparing other conventional therapies and has synergic effect with conventional therapies in treating IBS.

## 2 Methods

### 2.1 Study design

The protocol was designed according to Preferred Reporting Items for Systematic Reviews and Meta-Analysis Protocol (PRISMA-P) 2015 statement guidelines.^[25]^

### 2.2 Ethics

No ethical approvalis required since there is no personal information collection and patient recruitment.

### 2.3 Study registration

The protocol was registered in Research Registry (Registration number: reviewregistry986).

### 2.4 Eligibility criteria

#### 2.4.1 Participants

Patients who were diagnosed as IBS according to Rome criteria I to IV will be included. The Rome criteria is the most widely used criteria for functional gastrointestinal disorders due to its reliable diagnostic accuracy, practicability, and specified IBS subtypes.^[26]^ According to the Rome criteria IV, IBS could be diagnosed if the criteria below are fulfilled for the last 3 months with symptom onset at least 6 months prior to diagnosis; Recurrent abdominal pain on average at least 1 day/week in the last 3 months, associated with 2 or more of the following criteria:

1. Related to defecation,
2. associated with a change in the frequency of stool, and
3. associated with a change in the form of stool.^[27]^

All subtypes of IBS patient are eligible for this review. There is no restriction on participants’ age, gender, and human race.

### 2.5 Types of interventions

Studies on the effects of XYS or modified XYS (herbs-added prescription) will be considered eligible. XYS should be orally administrated with minimum 2 weeks of duration. There is no limitation about the dosage and the formation including pill or capsule, powder, granule, and decoction.

### 2.6 Types of comparators

There is no limitation for comparators. The conventional treatments and placebo or no treatments will be considered eligible. If the experimental group used combination therapy, comparators should be in consistent with the experimental group except XYS.

### 2.7 Type of studies

This SR includes published studies of randomized controlled trials (RCTs). Studies except RCTs including laboratory studies, case reports, cohort studies, review, and commentaries will be excluded. Cross-over trials which did not present the outcome measurements before the wash out will be excluded in order to prevent carry-over effect. Studies that did not provide the methods or conducted incorrect randomization will not be included. There will be no limits on the publication language.

### 2.8 Outcome measures

Efficacy rate will be the primary outcome measure. Secondary outcomes will include IBS-related indices (abdominal pain score, abdominal distension score, diarrhea or constipation score, bowel symptom severity scale), quality of life, and adverse events.

### 2.9 Information sources and search strategy

#### 2.9.1 Electronic searches

The following electronic databases will be used for searching studies: PubMed, Cochrane library, Excerpta Medica database (EMBASE), Oriental Medicine Advanced Searching Integrated System, National Digital Science Library, Korean Studies Information Service System, China National Knowledge Infrastructure, Wanfang databases, CiNii. Studies published up to April 2021 will be included.

Researchers will use search terms with a combination of diagnoses (irritable bowel syndrome, irritable bowel, irritable colitis, mucous colitis, allergic colitis, functional bowel disease, “IBS”) and treatments (XiaoYao, SoYo, ShoYo, traditional Chinese herbal formula). The example of search strategy for EMBASE is shown in Table 1. (Table 1).

**Table 1:** Search strategy for the EMBASE.

No.	Search terms
#1	“irritable bowel syndrome”/exp OR “irritable bowel syndrome” OR IBS OR “irritable colon OR ’functional bowel disease”
#2	allergic AND (“colitis”/exp OR colitis)
#3	mucous colitis
#4	#1 OR #2 OR #3
#5	“xiaoyao powder” OR “xiaoyaosan” OR “xiaoyao san” OR “xiaoyao” OR soyosan OR shoyosan
#6	“Chinese medicinal formula”/exp
#7	Chinese herbal formula
#8	Chinese medicinal herb
#9	#5 OR #6 OR #7 OR #8
#10	random∗ OR crossover∗
#11	“clinical trial”
#12	“randomized controlled trial”
#13	“single blind procedure”
#14	“double blind procedure”
#15	“crossover procedure”
#16	#10 OR #11 OR #12 OR #13 OR #14 OR #15
#17	#4 AND #9 AND #16

EMBASE = Excerpta Medica database

#### 2.9.2 Searching other resources

We will manually search for additional studies in reference lists of retrieved articles. We will also try to contact to author e-mail if needed.

### 2.10 Data collection and analysis

#### 2.10.1 Study selection

**Figure 1:**
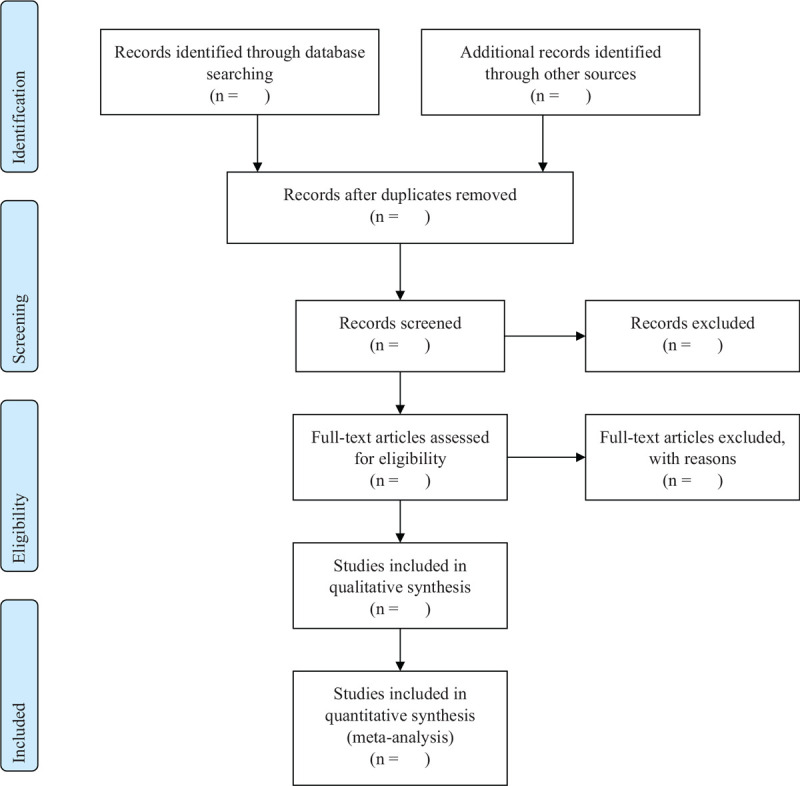
PRISMA flow diagram.

After briefing about the designed qualification, 2 researchers will conduct screening and reviewing for the eligibility. Studies will be screened by the title and abstract, and full-text (if needed). After excluding the duplicates and irrelevant reports, screened studies will be reviewed according to inclusion/exclusion criteria. Disagreement between the 2 researchers will be resolved through discussion and any dissenting opinions will be resolved by third reviewer (Fig. 1).

#### 2.10.2 Data management

Studies will be managed by EndNote X9.

#### 2.10.3 Data extraction

Data extraction will be conducted independently by 2 researchers and third party will check the data. Any disagreements will be resolved through discussion among 3 reviewers mentioned above. The following data will be extracted:

First author's name, year of publication, study design, characteristics of the patients, details of intervention given to each group, outcome measures, results, and information related to study quality.

For incomplete or obscure data, additional information or clarification will be requested by contacting corresponding authors. If there is no response, we will include only the available data and describe the reason in the paper.

#### 2.10.4 Data synthesis and analysis

The changes from baseline in the included RCTs will be combined using random-effect model or fixed-effect model, and Review Manager software (Version5.3; Copenhagen; The Nordic Cochrane Center, The Cochrane Collaboration, 2014) will be used to perform meta-analysis. The mean difference with 95% confidence intervals in the same outcome measure, and the standardized mean difference with 95% confidence intervals in the different outcome measure will be calculated to estimate the effect.

Heterogeneity will be determined by *Q*^*2*^ and *I*^*2*^ statistic according to the recommendations of the Cochrane handbook for systematic reviews of interventions.^[28]^ Interpretation of heterogeneity is as followed:

*I*^*2*^ statistic: 0% to 40% unimportant heterogeneity; 30% to 60% moderate heterogeneity; 50% to 90% substantial heterogeneity; and 75% to 100% considerable heterogeneity.
Cochran Q statistic (Q^2^*)*: *P* < .10 statistically significant.

If needed, meta-regression or sensitivity analysis will be conducted to find potential variables. If possible, subgroup analyses will be performed according to various criteria (e.g., IBS-subtype) and control interventions. When quantitative synthesis is not possible, narrative synthesis will be performed based on the available data.

Funnel plot will also be applied for assessment of reporting bias if more than 10 trials are included in the study.^[29]^ The Grades of Recommendation, Assessment, Development, and Evaluation will be utilized to rate the quality of evidence.^[30]^

### 2.11 Risk of bias assessment

Two reviewers will independently assess the risk of bias for the selected studies by using Cochrane Collaboration “risk of bias” tool^[28]^ and dissenting opinion will be resolved through discussion. Risk of bias will be conducted according to 7 domains:

1. Sequence generation.
2. Allocation concealment
3. Blinding of participants and investigators
4. Blinding of outcome assessment.
5. Incomplete outcome data
6. Selective reporting
7. Other biases.

## 3 Discussion

IBS is a disorder that affects patient's quality of life by not fully understood pathophysiology. While the conventional medical treatments do not fully satisfy the expected therapeutic effect, there have been reports about the clinical effectiveness of Chinese herbal formula therapy for IBS. Nonetheless, TCM still lack of scientific evidence for its clinical application on IBS, and there are no reviews which verified the usage of XYS for IBS. This review will suggest the clinical evidence of XYS effectiveness and safety for IBS and we hope our review can further support the further research on IBS.

## Author contributions

**Conceptualization:** Ji Ho Lee, Young Woo Kim.

**Funding acquisition:** Young Woo Kim.

**Investigation:** Ji Ho Lee, Won-Suk Sung.

**Methodology:** Won-Suk Sung.

**Project administration:** Eun-Jung Kim, Young Woo Kim.

**Supervision:** Eun-Jung Kim, Young Woo Kim.

**Writing – original draft:** Ji Ho Lee, Young Woo Kim.

**Writing – review & editing:** Won-Suk Sung, Eun-Jung Kim.
